# Expression of Immune Regulatory Genes in the Porcine Internal Genital Tract Is Differentially Triggered by Spermatozoa and Seminal Plasma

**DOI:** 10.3390/ijms20030513

**Published:** 2019-01-25

**Authors:** Manuel Alvarez-Rodriguez, Mohammad Atikuzzaman, Heli Venhoranta, Dominic Wright, Heriberto Rodriguez-Martinez

**Affiliations:** 1 Department of Clinical and Experimental Medicine, Faculty of Medicine and Health Sciences, Linköping University, SE-58185 Linköping, Sweden; manuel.alvarez-rodriguez@liu.se (M.A.-R.); atik.st@sau.ac.bd (M.A.); heli.venhoranta@googlemail.com (H.V.); 2 Department of Surgery and Theriogenology, Faculty of Veterinary Animal and Biomedical Sciences, Sylhet Agricultural University, 3100 Sylhet, Bangladesh; 3 Department of Physics, Chemistry and Biology, Faculty of Science and Engineering; Linköping University, SE-58183 Linköping, Sweden; dominic.wright@liu.se

**Keywords:** transcriptomics, microarray, bioinformatics, spermatozoa, seminal plasma, immune-regulation, female internal genitalia, pig

## Abstract

Mating or cervical deposition of spermatozoa or seminal plasma (SP) modifies the expression of genes affecting local immune defense processes at the oviductal sperm reservoir in animals with internal fertilization, frequently by down-regulation. Such responses may occur alongside sperm transport to or even beyond the reservoir. Here, immune-related gene expression was explored with cDNA microarrays on porcine cervix-to-infundibulum tissues, pre-/peri-ovulation. Samples were collected 24 h post-mating or cervical deposition of sperm-peak spermatozoa or SP (from the sperm-peak fraction or the whole ejaculate). All treatments of this interventional study affected gene expression. The concerted action of spermatozoa and SP down-regulated chemokine and cytokine (P00031), interferon-gamma signaling (P00035), and JAK/STAT (P00038) pathways in segments up to the sperm reservoir (utero-tubal junction (UTJ)/isthmus). Spermatozoa in the vanguard sperm-peak fraction (P1-AI), uniquely displayed an up-regulatory effect on these pathways in the ampulla and infundibulum. Sperm-free SP, on the other hand, did not lead to major effects on gene expression, despite the clinical notion that SP mitigates reactivity by the female immune system after mating or artificial insemination.

## 1. Introduction

Semen is a complex suspension of cells (mostly spermatozoa as well as spermatogenic cells, epithelia from the genital tract or invading immune cells) in a complex fluid; the seminal plasma (SP). Semen is recognized as foreign by the female immediately after insemination. In the porcine species, 60–120 billion spermatozoa are ejaculated at one time, suspended in a 150–250 mL ejaculate volume. This is sequentially deposited into the cervix of the female during natural mating [1]. Despite the large spermatocrit, only a small sperm subpopulation (10^5^–10^8^) reaches the functional sperm reservoirs (utero-tubal junctions, UTJ) in the oviducts within 5–60 min of insemination [1,2]. Stored spermatozoa are alive and functionally fit during the pre-ovulatory period, before being gradually released for fertilization in relation to ovulations [3]. However, most of the inseminated spermatozoa do not reach the UTJs. Within 30 minutes of insemination about 20–25% of the spermatozoa are egressed by vaginal retrograde flow [4,5] while those spermatozoa remaining in utero are phagocytosed by polymorphonuclear leukocytes (PMNs) migrating from the endometrial lamina propria [1,6,7,8,9]. Interestingly, such an inflammatory scenario is not seen in the pre-ovulatory UTJs [10,11], and it is normally transient (over a matter of hours) in the uterus [11]. 

Entry of semen into the sow genitalia leads to a brief uterine inflammation. It also seems to change the nature of the innate and acquired local immunity in the female reproductive tract after entry [1,7,9,12]. Such an effect ought to be initiated by signaling originating from the spermatozoa as well as from the complex SP and its battery of proteins, peptides and enzymes [13,14,15,16,17], cytokines/chemokines [18,19,20], sex hormones [21,22], extracellular vesicles (i.e., exosomes), lipoproteins and non-coding RNAs [23,24,25]. At present, however, our knowledge of the genetic and physiological pathways that cause these changes in the immune system of the female are poorly understood. 

For such sustained changes, no matter what the initiating molecule or the responsible pathway is, specific gene expression changes must be activated in the female genitalia. For instance, the presence of spermatozoa in the murine oviduct leads to changes in gene expression, with the up-regulation of adrenomedullin and prostaglandin-endoperoxide synthase 2 transcripts [26]. Similarly, the pig oviduct shows changes in the expression of a diverse set of genes [27,28,29]. Focusing on the tubal sperm reservoirs in chicken, we have previously demonstrated that mating changed gene expression in the sperm reservoir of an advanced inter-cross line between Red Junglefowl and White Leghorn chickens [30]. Insemination has been shown to increase mRNA expression of transforming growth factor beta (*TGFβs*) genes and TGFβ receptors (*TβRs*) and to decrease mRNA expression of interleukin 1 beta (*IL1B*) and of lipopolysaccharide induced tumor necrosis factor TNF (*LITAF*) in the sperm-storage tubuli (SST). These changes have been implicated in the survival of spermatozoa in the sperm reservoir [31,32,33]. Interestingly, both mating and the infusion of sperm-free seminal fluid elicit the activation of genes involved in pH-regulation (*SLC16A2*, *SLC4A9*, *SLC13A1*, *SLC35F1*, *ATP8B3*, *ATP13A3*) or immune-modulation (*IFIT5*, *IFI16*, *MMP27*, *ADAMTS3*, *MMP3*, *MMP12*) in the sperm reservoirs of both chicken and pigs, generally being down-regulated in both animal classes [34].

In pigs, semen is ejaculated in sequential fractions, classically defined as the pre-sperm fraction, the sperm-rich fraction (SRF) and the post-SRF [1]. The first 10 mL portion of the SRF (P1) is the sperm-peak fraction, holding 25% of the total ejaculated spermatozoa, as well as a protein-poor specific part of the SP (the cauda epidydimal fluid and secretion from the prostate) [1]. The post-SRF is highly rich in proteins, owing to dominating secretions from the seminal vesicles [16], so it can potentially elicit distinct gene expression changes during sperm transport. These changes can occur in the sperm reservoir, or even in upper segments such as the ampullary-isthmic junction (AIJ), where fertilization usually occurs [29]. The study presented here aims to determine pre/peri-ovulation expression changes of immune-related genes in tissues collected from different segments of the internal female genital tract (cervix to infundibulum). Tissues were collected 24 h after either (i) mating (entire semen deposition), (ii) cervical insemination of P1-semen extended in Beltsville Thawing Solution (BTS) or (iii) cervical deposition of sperm-free SP from the P1-fraction of the ejaculate, or of the (iv) whole SP (from the entire ejaculate) and assayed against untreated controls. Given the role of the immune system in our previous work on similar experiments in chicken, we have focused solely on genes with known immune functions.

## 2. Results

Given the lack of side differences for uterine horns and oviducts in a preliminary analysis (see Section 4.2 Materials and Methods), only right-hand side tissue samples were used in this study.

### 2.1. Entry of Semen-Induced Expression Changes of Genes Involved in Immune System Processes in the Entire Female Genital Tract

**Figure 1:**
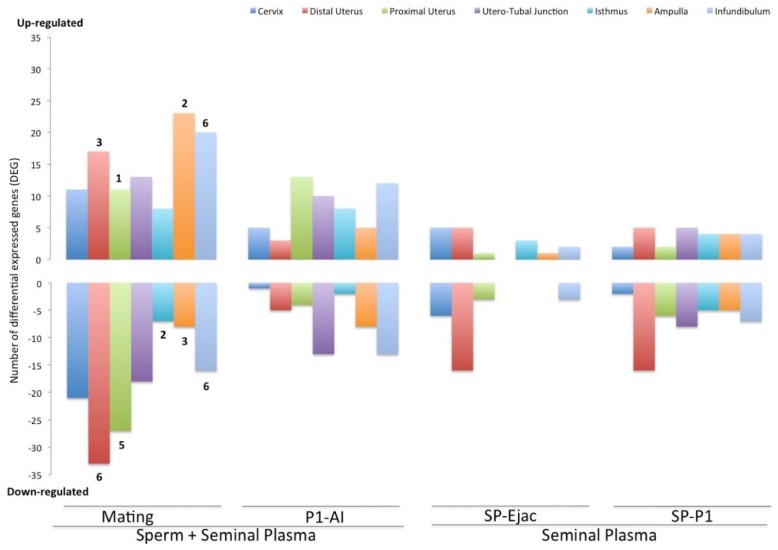
Distribution of differentially expressed annotated genes belonging to pathways of the immune function process (GO:0002376), up- and down-regulated (*p* < 0.05) along segments of the internal female genital tract (Cervix to Infundibulum) after the different treatments (Mating: sow mated with a boar; P1-AI: sow artificially inseminated with the sperm-peak portion (P1) extended to 50 mL with Beltsville Thawing Solution (BTS); SP-Ejac: sow cervically infused with sperm-free SP of the whole ejaculate (50 mL); SP-P1: sow cervically infused with sperm-free SP from pooled sperm-peak portion P1 (50 mL). All treatments were compared to Control (cervical infusion with 50 mL of BTS). The numbers of false discovery rate (FDR)-corrected (*q*-value < 0.05) genes are presented over/under bars in the mating treatment.

Figure 1 displays the relative numbers of annotated genes belonging to pathways of the immune function process (GO:0002376) that were differentially expressed (*p* < 0.05) in the different ascending segments of the sow genital tract (Cervix to Infundibulum), per treatment. Mating was the treatment, combining all tissues, with the highest effect on these immune-related genes, even when compared to the P1-AI treatment. Please note that significance using a false discovery rate (FDR)-corrected threshold (*q*-value < 0.05) was only found for genes in the mating treatment (numbers in the graph of Figure 1). Of the 154 annotated immune-related genes (excluding repeating genes in all tissues), 117 of them (65%) were up- or down-regulated (*p* < 0.05) 24 h after treatment, as compared to controls. Comparing the presence of semen (entire ejaculate or only the P1-fraction) with the sperm-free SP-infusions, it was evident that the latter induced the expression of fewer genes (35 genes differentially expressed in SP-Ejac (17 up-regulated and 28 down-regulated) and 75 genes differentially expressed in SP-P1 (26 up-regulated and 49 down-regulated)). Of note, more genes were down-regulated than up-regulated up to the UTJ (130 vs. 103) when mating was involved, but not when only the sperm-peak fraction (P1-AI) was used. The situation when sperm-free infusions were performed was similar to mating (more down-regulated genes). Of interest, infusion of SP from the entire ejaculate (SP-Ejac) was neither able to modify the expression of any immune-related genes in the UTJ, nor to down-regulate genes in the adjacent isthmic or ampullar segments (see Figure 1). Infusion of only the SP-P1 fraction was, on the other hand, able to modify expression (UTJ: 5 up-regulated and 8 down-regulated; Isthmus: 4 up-regulated and 5 down-regulated; Ampulla: 4 up-regulated and 5 down-regulated).

Following the Principal Component Analysis (PCA) analysis of the data (see M&M and the Supplementary Table S1), 49 genes were differentially expressed after mating (16 at the *p* < 0.003 PCA threshold) and 33 genes were differentially expressed after P1-AI (11 at the *p* < 0.003 PCA threshold). The numbers of differentially expressed genes were much lower in the sperm-free SP treatments, with only 4 genes differentially expressed after SP-Ejac exposure (only one (1) at the *p* < 0.003 PCA threshold), and 7 genes differentially expressed following SP-P1 exposure (5 at the *p* < 0.003 PCA threshold) (Supplementary Table S1).

**Figure 2:**
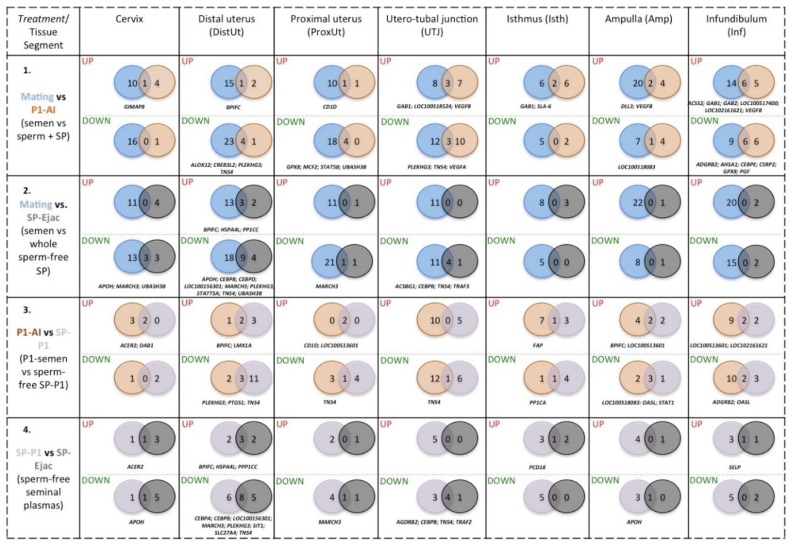
Venn diagrams displaying numbers of differentially expressed genes of immune function (up- or down-regulated, *p* < 0.05) in the internal genital tract of sows, comparing the various treatments used (1). Semen (spermatozoa and SP) from the entire ejaculate (Mating) or the sperm-rich fraction (P1-AI); (2). Semen (spermatozoa and SP) from the entire ejaculate (Mating) or its sperm-free entire SP (SP-Ejac); (3). Semen (spermatozoa and SP) from the sperm-peak fraction (P1-AI) or its sperm-free SP (SP-P1); SP (sperm-free) from the sperm-peak fraction (SP-P1) or from the whole ejaculate (SP-Ejac). The acronyms of genes common to treatment per tissue (crossing sectors) are identified.

A series of Venn diagrams are presented in Figure 2 displaying the numbers of differentially expressed genes of immune function (up- or down-regulated, *p* < 0.05) in the internal genital tract of sows comparing combinations of treatments. As well, the diagrams indicated which genes were identified as common to treatment per tissue. The effect of semen (spermatozoa and the accompanying SP) was comparable between Mating (a complete ejaculate) and the P1-AI (Figure 2, comparison of row 1). This suggests even just the entry of the P1 fraction of the ejaculate (Comparison 1) affected gene expression over the entire length of the female genital tract. There was a tendency for the most common differentially expressed genes to be down-regulated (18 vs. 16). In addition, the tissues with the highest numbers of differentially expressed genes common to multiple tissue types were the UTJ (15 genes) and the Infundibulum (17 genes). Comparisons between sperm-containing treatments and the sperm-free SP treatment (rows 2 and 3 respectively, Figure 2) demonstrated a large variation in the numbers of common differentially expressed genes. Generally, more genes were down-regulated after mating than by sperm-free SP (17 vs. 3). This can be seen in the comparison of mating versus sperm-free SP treatments, whole ejaculate SP vs sperm-peak P1 fraction treatments (16 vs. 6), but not within the P1 fraction treatment (11 vs. 11), see Figure 2. However, none of these down-regulated genes were common to all treatments, except for certain genes that were down-regulated in the UTJ and DistUt (Figure 2, row 2). Very few genes were differentially expressed in the upper oviductal segments, e.g., beyond the UTJ; effects were generally only observed in the uterus (ProxUt and DistUt) and the UTJ. Notably, there were essentially no effects of the SP in the oviductal segments, Isthmus-to-Infundibulum.

### 2.2. Gene Ontologies of the Immune Functions Hit by the Differentially Expressed Genes 

The bioinformatics analysis showed that several differentially expressed genes in various segments of the female genitalia elicited action via immune-process pathways (GO:0002376)**.** Supplementary Table S2 summarizes the number of differentially expressed genes belonging to immune processes found in the different segments of the sow genital tract 24 h after the various treatments, displayed different pathways, up- or down-regulated (*p* < 0.05, red: FDR, *q* < 0.05), with the full list of these genes presented in Table 1. Of particular relevance is the inflammatory action that mating would have exerted via the Chemokine and Cytokine pathway (P00031) in all segments but the UTJ or the adjacent isthmus. Other specific immune-related pathways that were present were the Interleukin signaling (P00036) and the JAK/STAT (P00038) pathways, which were down-regulated by mating and up-regulated by the SP of the P1 in the UTJ. B-cell (P000010) and T-cell (P00053) activation pathways were also present in the UTJ (down-regulation after P1-fraction semen entry). Up to 14 differentially expressed genes involved in immune system processes were present more than once (see Supplementary Tables S1 and S2 and Table 1). Table 2 lists the PCA (*p* < 0.003) and FDR-corrected (*q* < 0.05) genes related to immune function, including their Kyoto Encyclopedia of Genes and Genomes (KEGG) pathway/s, displaying their changes in expression along the pig estrous internal genital tract, 24 h after mating or cervical deposition of the sperm-peak portion (P1-AI), of its SP (SP-P1) or the SP from the whole ejaculate (SP-Ejac). Please note that in this table we cannot say whether these pathways are enriched relative to other non-immune-related pathways, as only immune genes were analyzed in this data. Rather, this table identifies which specific type of immune genes are enriched in the various tissues analyzed. 

## 3. Discussion

Mating (whole ejaculate) and cervical deposition of a sperm-bearing specific vanguard portion of the ejaculate (P1), and of sperm-free SP from the same ejaculate components were able to elicit expression changes in genes related to immune processes at different segments of the female internal genital tract in sows, 24 h post-exposure. The influence of semen on gene expression changes confirmed previous studies in other species [26,35] and under different experimental designs in a porcine model [27,28,29]. 

A priori, the entry of the sperm-free SP component induced a weaker response than the whole semen (spermatozoa + SP) component, indicating that exposure to spermatozoa played a major role in the induction of gene expression. Exposure to SP from the entire ejaculate mimicked the down-regulatory effects observed after the standard mating treatment, while exposure to the SP accompanying spermatozoa in the P1-sperm-peak vanguard portion of the ejaculate caused up-regulation of specific genes. These results indicate a concerted signaling induction by spermatozoa and the surrounding SP, mainly acting to down-regulate genes related to immune pathways. Such signaling would decrease the capacity of the female immune system to reject those spermatozoa stored in specific genital tract compartments, and is a probable mechanism behind the so-called maternal immune tolerance effect [12].

When spermatozoa from the sperm-peak (P1) fraction was extended with BTS and cervically deposited, mimicking AI, the effect was different from standard mating. This treatment led to the up-regulation of specific genes (*GAB1*, *BPIFC*, *CD244*, *WFIKKNZ*). Whether this is related to an up-regulatory sperm-effect, considering the P1-AI treatment had a diluted SP; or related to the specifics of this particular sperm-peak fraction is yet to be determined. However, the SP as a fluid is mostly bathing the uterine lumen, and it is still questionable whether it enters the UTJ-sperm reservoir or the upper oviduct segments i.e., isthmus-to-infundibulum [5]. However, it has been experimentally proven that relevant SP-molecules are adsorbed to the plasma membrane of the spermatozoa at ejaculation and travel to the UTJ [36]. Some of these molecules are lost during capacitation [1,37] but some, such as the spermadhesin AWN-1, remain attached to the sperm plasmalemma up to the site of fertilization [38]. In this context, these data would mean that the sperm-SP ought to be considered a discrete physiological entity potentially triggering gene expression upon contact with the cells lining the internal genital tract of the female. Logically, it would explain why the response was dissimilar between sperm-SP and sperm-free fluids.

In a previous study, comparing seminal responsiveness by the female tubal reservoirs in chicken and pigs, sperm-presence also elicited a major response of genes related to sperm viability, including immune modulatory-genes, conserved over these animal classes [34]. Therefore, in this study just genes related to the immune system were focused upon. These genes were chosen based on the PANTHER GO-category (GO:0002376) term ‘immune system process’ and comprised a total of 154 genes. To control for multiple testing correction, we used two separate methods, an FDR-based correction and a corrected *p*-value based on the substructure present in these immune system genes [39], see Materials and Methods. The PCA method gives a significance threshold of *p* < 0.003 and a suggestive threshold of *p* < 0.01, taking onto account the substructure present within the immune-related genes. Of the 154 immune-related genes, 93 were either suggestive or significantly differentially expressed in one or more treatments. Most of these genes were expressed after mating (49 genes at a nominal 0.05 level, 16 at the *p* < 0.003 PCA threshold), followed by P1-AI (33 genes at a nominal 0.05 level, 11 at the *p* < 0.003 PCA threshold), the SP-Ejac (4 genes, one (1) at the *p* < 0.003 PCA threshold) and the SP-P1 (7 genes at a nominal 0.05 level, 5 at the *p* < 0.003 PCA threshold). These results confirm the overall distribution, and further allowed to focus on the type of response, with mating and sperm-peak fraction (P1-AI) affecting gene expression over the entire length of the internal genital tract. Of interest was the finding that sperm-free SP treatments barely affected differential expression of genes at the ad-ovarian segments, which confirms the exposure to SP is low in these tract segments [5]. Mating induced a general down-regulation of gene expression (39/49) while P1-AI induced a general up-regulation (21/33) of genes involved in immune response processes. This difference in responsiveness between mating and P1-AI is particularly interesting, given that the treatments are similar, besides the fact that mating involves a full physiological reproductive event. Mating implied the entry of all spermatozoa and the entire contents of the SP, while P1-AI consisted of only the sperm-peak fraction of the ejaculated spermatozoa surrounded by a diluted distinct fraction of the SP, essentially composed of epididymal cauda fluid and prostate secretion [1,40,41]. Regarding the SP-effects, despite the number of differentially expressed genes being much lower as compared to the mating/P1-AI treatments, the SP-Ejac treatment induced a general down-regulation of immune genes (3/4) while the SP-P1 treatment induced a general up-regulation of genes involved in immune response processes (6/7), thus mimicking the sperm-bearing treatments. Consequently, it appears that the bulk of the SP, ejaculated after the SRF, might be the principle agent influencing the down-regulation of immune genes. This post-SRF has an enormous amount of specific protein and peptides, 10–20-fold higher than the P1-portion, derived from the secretion of the seminal vesicles [16]. Whether these SP-components are responsible for the alteration of immune-related genes is, however, yet to be established.

Considering the role of the SP in initiating the immune response, it also appears that these responses are largely confined to the lower genital tract segments (57 differentially expressed genes in the lower tract vs 36 in the upper tract). The observed data potentially indicate that the induction of expression changes relate to spermatozoa being present in significant numbers in the lower segments and/or that the SP did not enter the upper ones. The latter conclusion is relevant since sperm-adsorbed SP-components are only seemingly present in the upper segments, while the lower ones are fully exposed to both spermatozoa and SP.

Regarding the sperm reservoir, where a subpopulation of potentially fertile, live spermatozoa is stored in sufficient numbers for fertilization of the ovulated oocytes for up to 40 h [2,42], the relative distribution of gene expression followed the same overall pattern in the various treatments. Mating and the sperm-peak vanguard treatments caused down-regulation, while only a weak effect of the sperm-free SP treatments was observed. It has been shown that cells involved in innate immunity are present in the uterine lumen from 30 minutes after the entry of semen or SP into the female tract and are sustained there for several hours. In contrast, such cells are largely absent from the sperm reservoir [11,43]. In terms of what specific immune genes and pathways are affected in these tissues, mating and P1-AI treatments down-regulated genes involved in the chemokine and cytokine pathway (P00031), the interferon-gamma signaling pathway (P00035) and the JAK/STAT (P00038) pathway in all segments of the internal genital tract up to the sperm reservoir (UTJ/isthmus). These treatments also up-regulated genes involved in these same pathways in the ampulla and infundibulum. Both mating and P1-AI treatments down-regulated the Interleukin signaling pathway (P00036) in all segments but the SP-P1 treatment up-regulated genes involved in these pathways in the UTJ. Genes affecting the B-cell (P000010), T-cell (P00053) and Wnt-signaling (P00057) activation pathways were also represented in the DistUt and ProxUt (down-regulated after mating), UTJ (down-regulation after P1-AI) and Isth (down-regulation after SP-P1). Gene ontologies of the differentially expressed genes shows most immune responsive pathways involved cytokines, T cells, and cells affecting innate immunity, while B-cell-mediated pathways did not appear to be present in any of the segments of the female tract.

Earlier studies stated that pig oviductal sperm reservoirs (UTJ/isthmus) do not show signs of infiltration by immune cells nor display other obvious immune responses during the pre-ovulatory period [10,11]. In utero, by contrast, large numbers of cervically inseminated spermatozoa are lost due to inflammation-related leukocyte phagocytosis and CD4–CD8 lymphocyte attacks [1,6,7,8,9,43]. Therefore, the female immediately reacts to the entry of semen with a transient, hour-lasting inflammation, where polymorphonuclear leukocytes are seen traversing the genital epithelium at the cervix and uterus to engulf spermatozoa as well as eliminating possible pathogens that accompany the non-sterile semen [1,9,44]. This inflammation is rapidly established in sows. The first leukocytes enter the uterine lumen 10 minutes post-mating/AI, with these cells peaking in number after about 30 minutes, and being sustained for 3–6 h before declining [1,13]. Similar findings have been seen in the upper oviduct (isthmus/ampulla) and particularly in the infundibulum, where the lining epithelium is continuous with that of the peritoneum [45]. SP proteins, often derived from the seminal vesicles, are seemingly responsible for these responses, and accompany the recruitment of cytokines and chemokines in the female genital tract in many species, including pig [13,46,47].

A further finding in the present study is that both the mating and insemination of the P1-sperm-peak fraction treatments were able to upregulate genes relating to trans-epithelial leukocyte infiltration after bacterial invasion (*VAV2*, [48]) and phagocytosis (FC epsilon and gamma, *GIMAP6*, [49,50]) in the UTJ, where leukocyte infiltration is not seen [13,42]. Down-regulation of *GAB1* and *GAB2* genes, linked to the transmigration of leukocytes after bacterial presence [51], were also seen in the UTJ. In the infundibulum, however, genes related to natural killer cytotoxicity and apoptosis were down-regulated (*PGF*) while *TNFSF10* was up-regulated. In contrast, TNFSF11 appeared to be down-regulated in the UTJ [52,53,54]. The findings of the present study indicate that the female genital tract responds to the entry of semen by altering genes related to the elimination of microorganisms. That these effects were detected in the upper genital tract segments but not in the cervix nor uterus might well reflect the timing of the tissue collection, considering immediate protective actions are transitory, and might have resumed by the time sampling occurred.

The cervix and uterus responded strongly to the mating treatment, which caused a plethora of genes to down-regulate (Cervix: *CD209*, related to lectin-receptors in phagosomes [55]; *UBASH3B*, related to cell membrane stability, control of cholesterol turnover [56]; Uterus: *UBASH3B*, *APOH* [57], *GPX5/GPX8*, coding for antioxidant enzymes related to membrane preservation, [58]) and *FAP*, related to the JAK-STAT pathway controlling cytokine function [59]). Mating was also able to down-regulate both *TNFSF11* and *ADGRB2* in the UTJ and ampulla segments, suggesting a down-regulation of cytokine actions [52] that might promote the development of a tolerance towards spermatozoa in the oviduct. The gene *VAV2* was also found to be down-regulated by the P1-AI treatment in the UTJ, which is in line with the development of an immune tolerance in the reservoir [48]. In addition, the P1-AI treatment caused an up-regulation of the *WFIKKN2* gene, which encodes for the multidomain proteins WFIKKN1 and WFIKKN2, regulating the transforming growth factor beta (TGFB) superfamily [60]. The chemokine TGF-ß is a modulator of maternal immune tolerance in several species, including pigs [61,62,63].

All treatments in the present study, besides the control (BTS), contained some level of SP, with or without spermatozoa. The SP is rich in immune regulatory proteins, peptides, and cytokines [13,16,64]. Specific SP proteins such as PSP-I and PSP-II can induce inflammation responses and changes in CD4+ and CD8+ lymphocytes [13]. It has been reported that boar SP could recruit cytokines, T cells and leukocytes relating to innate immunity [45,65,66], including action mediated by exosomes onto endometrial epithelium [67]. However, insemination of sperm-free SP provided evidence of very few genes being affected. In particular, only the P1-AI treatment was able to induce up-regulation of *HSPA4L*, *DPP4* and *APOH* in the uterus and isthmus/ampulla. 

In the present study, mating has been considered as a positive in vivo control. During mating there is a mechanical stimulation of the cervix and the deposition of a large volume of semen, in sequential fractions [1]. The complex SP contains many immune-related proteins, cytokines [13,16,20] and antioxidant enzymes such as GPX-5, paraoxonases and catalase [14] all considered to play an important role in sow fertility [64,68]. Since the sperm-peak fraction of the boar ejaculate is physiologically different from the other fractions, with greater sperm numbers and different biochemical properties [40,41], we have also infused semen from the sperm-peak fraction (containing spermatozoa and its surrounding SP fraction, rich in prostate secretion and most of the cauda epididymal fluid). However, the effects of this treatment clearly differed from the positive control mating, perhaps in relation to the different synergy provided by the association of SP proteins and spermatozoa, a fact we have demonstrated previously [17].

The current results are only based on RNA expression, and as such further work needs to be performed to ascertain whether these changes in RNA expression translate to differences in protein levels, as well as the duration that these changes in gene expression exist for. Such protein abundances, production, and turnover can have large effects, though many open questions remain regarding the specificity of translation feedback and regulation [69]. As such, the results shown here represent an important first step in elucidating the role of the immune system and the genetic basis of the changes that occur during fertilization, but more work remains to be done.

## 4. Materials and Methods 

### 4.1. Ethics Statement of the Interventional Study

Animal husbandry and experimental handling were performed in compliance with the European Community (Directive 2010/63/EU, 22/09/2010) and current Swedish legislation (SJVFS 2017:40). The experiments were approved in advance by the “Regional Committee for Ethical Approval of Animal Experiments” (Linköpings Djurförsöksetiska nämnd) in Linköping, Sweden (permits no. 75-12 (10/02/2012) and no. ID1400, 02/02/2018). 

### 4.2. Experimental Design

A preliminary gene expression analysis (data not shown) of eventual differences between selected samples from the right vs left sides of the tubular genital tract (uterine horns and oviducts) yield no significant differences between sides. Gene expression analyses were therefore fully performed in female internal genital tract segments (endocervix (Cvx) and the right side of the tract e.g., distal uterus (DistUt), proximal uterus (ProxUt), UTJ, isthmus (Isth), ampulla (Amp) and infundibulum (Inf)).

The segments were surgically retrieved under general anesthesia 24 h after treatment in 20 domestic female pigs (*Sus scrofa domestica*). The females were allotted in equal numbers (*n* = 4) to one of five separate groups:

1. Control (*n* = 4), control group, where females were cervically infused with the protein-free extender BTS (50 mL);
2. Mating (*n* = 4), where females were each mated with a single male;
3. P1-AI (*n* = 4), where females were artificially inseminated with ejaculated P1-sperm-peak fractions (extended to 50 mL with BTS);
4. SP-Ejac (*n* = 4), where females were cervically infused with sperm-free SP of the whole ejaculate (50 mL);
5. SP-P1 (*n* = 4), where females were cervically infused with sperm-free SP harvested from ejaculated sperm-peak fractions (P1, pool, 50 mL).

### 4.3. Animals and Tissue Sources

Young mature boars (9–11 mo, *n* = 5) of proven sperm quality (concentration, motility, and morphology) and weaned sows (parity 1–3, *n* = 20) of Swedish Landrace breed were recruited from a controlled breeding farm. Throughout all experiments, animals were handled carefully and in such a way as to avoid any unnecessary stress. The animals were individually kept in separate pens at the Translational Medicine Centre (TMC/CBR-3) of Linköping University under controlled temperature and light regimes (12 h:12 h light/dark cycle). Pigs were fed with commercial feedstuff (Lantmännen, Stockholm, Sweden) according to national standards [70], provided with water ad libitum and receiving the same management.

### 4.4. Semen Collection, Evaluation, and SP Harvesting

Semen was manually collected (gloved-hand method) weekly. All ejaculates/fractions collected were evaluated for sperm concentration and motility (velocity and forward progressive motility) using a light microscope (Zeiss, Stockholm, Sweden) equipped with a thermal plate (38 °C), positive phase contrast optics (10× objective), a Charge Coupled Device (CCD) camera (UI-1540LE-M-HQ, Ueye, IDS Imaging Development Systems GmbH, Obersulm, Germany), and the Qualisperm^®^ Software (Biophos SA, Lausanne, Switzerland). Ejaculates with at least 70% motile and 75% morphologically normal-looking spermatozoa immediately after collection were used for the experiments. The SP was harvested either from the whole ejaculate or from the sperm-peak P1-fraction after double centrifugation at 1500× *g* for 10 min, and checked for absence of spermatozoa. The harvested sperm-free crude SP was stored at −20 °C until used.

### 4.5. Handling of Sows

The females were observed two times daily for pro-estrus and estrus behavioral signs while holding snout contact with a neighboring boar and the application of back pressure by experienced personnel. Animals that showed a standing estrus reflex were considered to be on the first day of behavioral estrus and were used in the experiments, randomly allotted to **control** (insemination with BTS, 50 mL) or treatment groups (e.g., Mating, P1-AI, SP-Ejac, or SP-P1). All inseminations/infusions were done cervically using disposable conventional AI-catheters (Minitüb, Munich, Germany) 

### 4.6. Tissue Sample Preparation

On the second day of standing estrus (pre-/peri-ovulation), e.g., 24 h after mating/inseminations, the sows were sedated by the administration of a mixture of 5 mg dexmeditomedine (Dexdomitor, Orion Pharma Animal Health, Sollentuna, Sweden) and 100 mg tiletamine hydrochloride/zolazepam hydrochloride (Zoletil vet, Virbac A/S, Kolding, Denmark), intramuscularly. General anesthesia was induced using sodium thiopental (Abbot Scandinavia AB, Solna, Sweden), 7 mg/kg body weight, intravenously, and was maintained with isoflurane (3.5–5%, Baxter Medical AB, Kista, Sweden) administered via a tracheal cuffed tube by a close-circuit PVC-ventilator (Servo ventilator 900 D, SIEMENS-ELEMA AB, Solna, Sweden). Peripheral blood was collected using vacutainer containing K2EDTA (Vacuette^®^, Greiner Bio-One GmbH, Kremsmünster, Austria) and were centrifuged at 300× *g* for 10 min at room temperature. The blood plasma was harvested and stored at −20 °C until analyzed for estradiol (E_2_) and progesterone (P_4_) concentrations. The female genital tract segments retrieved immediately after clamping the irrigating blood vessels, and were plunged into liquid nitrogen (LN_2_) before being stored at –80°C except utero-tubal junction which was divided longitudinally—one half was stored at –80°C and other half was fixed in 4% paraformaldehyde for histological confirmation of presence or absence of spermatozoa. The ovaries were photographed to count the number of pre-ovulatory follicles or eventual new ovulations. There was an average of 22.30 ± 7.29 (mean ± standard deviation) pre-ovulatory follicles per sow, without significant differences between sow-groups, found.

### 4.7. Determination of Estradiol (E_2_) and Progesterone (P_4_) Concentrations

Concentrations of E_2_ and P_4_ were individually measured in blood plasma (50 μL) using a porcine specific enzyme linked immunosorbent assay (ELISA) kit (Cat# MBS700342 and Cat# MBS703577, MyBiosource Inc., San Diego, CA, USA), after preparation of a standard curve for each hormone, following the manufacturer protocol. The optical density of each microplate well was determined using a microplate reader (Tecan, Sunrise GmbH, Grödig, Austria) at 450 nm wave length. The E_2_ concentrations (mean ± SD pg/mL) were 349.10 ± 62.19 in Mating, 319.43 ± 86.42 in P1-AI, 294.20 ± 80.24 in SP-Ejac, 340.20 ± 13.95 in SP-P1 and 376.50 ± 27.76 in Control group sows, e.g., without significant differences. The P_4_ concentrations were found to be ≤0.77 ± 0.35 in all sows. The endocrine values confirmed the animals were in pre-/peri-ovulatory estrus when samples were retrieved for gene expression analyses.

### 4.8. Microarray Hybridization and Scanning

Total RNA was extracted using Trizol from tissue samples of each genital tract segment and quality checked following methods previously described [34]. Equal amounts of total RNA (250 ng) from each sample were used to make cDNA using GeneChip® Whole Transcript Plus reagent kit (Affymetrix, Santa Clara, CA, USA) following the manufacturer protocol. Finally, 3.5 μg of fragmented and labelled single stranded cDNA (41 μL) were mixed with 109 μL of hybridization master mix to make a cocktail hybridization mix for a single reaction. The hybridization cocktail was then incubated first at 99 °C for 5 min, followed by a descent to 45 °C until loaded on the array chip (GeneChip^®^ Porcine Gene 1.0 ST Array, Affymetrix Inc., 3420 Central Expressway, Santa Clara, CA 95051, USA). A total of 130 μL of the cocktail hybridization mix was loaded into each array chip and was incubated at 45 °C under 60 rotations per min, for 16 h. The hybridized cartridge array chip was then unloaded and subjected to washing and staining using a GeneChip^®^ Fluidics Station 450 (Affymetrix), to be finally scanned using the Affymetrix GeneChip^®^ scanner GCS3000.

### 4.9. Microarray Data Analysis and Bioinformatics

The intensity data of each array chip was processed using the robust multi-array average (RMA) normalization, computing average intensity values by background adjustment, quantile normalization among arrays and finally log_2_ transformation for extracting the expression values of each transcript in the probe set, as implemented in the official Transcriptome Analysis Console (TAC; version 4.0) from Affymetrix. In this experiment, only genes relating to the immune system were assessed in detail. These genes were identified as being those using the PANTHER Classification System for GO [71], with the 154 genes that fell under the “immune system processes” term (GO:0002376) being selected from the whole genome transcription data. The statistical analysis of the normalized gene expression data was performed using a linear model using the empirical Bayes’ approach as implemented in the package “limma” was used to calculate differentially expressed transcripts using a Benjamini-Hochberg FDR (*q* < 0.05) and a PCA-based p-value correction to control for multiple testing to control type-I errors [72]. Due to the relatively limited number of genes being assessed (154), the FDR correction does not accurately take onto account the correlational relationship between the different immune genes. With multiple testing correction, the number of independent tests, needs to be controlled for. To establish how many of the immune genes were correlated with one another (and therefore do not require full Bonferroni multiple testing correction) a PCA, [39]) was performed on the 154 immune genes with a statistical cut off *p* < 0.05 or FDR *q* < 0.05. This PCA analysis had 19 significant eigenvalues (>1) that explained all the variation in the data (100% for all tissues). Therefore, rather than a Bonferroni correction of 154, a correction of 19 was applied instead to account for the correlations between immune genes, resulting in a significance threshold of (*p* < 0.003), while a suggestive threshold of 20% gave a threshold of (0.2/ 19) = *p* < 0.01). Both redundant and uncharacterized transcripts were excluded from the initial list to make a final list of differentially expressed genes. The list of immune-related genes identified in PANTHER were then searched for functional pathways using the KEGG database [73]. The differentially expressed 154 genes (*p* < 0.05) were screened for additional molecular functions with the protein knowledge base of the UniProt Consortium [74] to confirm they were correctly classified as belonging to the immune system process.

## 5. Conclusions

The findings indicate that there is a concerted action of spermatozoa and SP affecting gene expression in the pig female genital tract towards down-regulation, 24 h after treatment, during the pre-/peri-ovulatory stage. Spermatozoa, at least those in the vanguard sperm-peak fraction, seem to have an up-regulatory effect, visible in the upper (oviductal) segments. Sperm-free SP, on the other hand, did not seem to play major effects on gene expression, despite the clinical notion that SP mitigates reactivity by the female immune system after mating/AI.

## Figures and Tables

**Table 1:** Genes belonging to immune processes, up- or down-regulated (*p* < 0.05, *red: FDR, q < 0.05*) found in the different segments of the sow genital tract (cervix to infundibulum), 24 h after mating or cervical deposition of the sperm-peak portion (P1-AI), of its SP (SP-P1) or SP from the whole ejaculate (SP-Ejac).

Treatment	Target	Up-Regulated Genes (log Fold Change, *p* < 0.05)	Down-Regulated Genes (log Fold Change, *p* < 0.05)
Mating	Cvx	*VEGFB*, *GAB1*, *TMEM131*, *CELSR1*, *DPP4*, *SPATA13*, *CDIP1*, *ACSL3*, *BPIFC*, *GIMAP8*, *CD244*	*STAT2*, *APOH*, *DLL4*, *FAP*, *GPX8*, *HSPA4*, *LITAF*, *LOC100518083*, *MARCH3*, *PLEKHG3*, *SELP*, *SLA-7*, *SLC11A1*, *STAT1*, *STAT5A*, *UBASH3B*
	DistUt	*GAB1*, *CD209*, *LOC102161621*, *WEE1*, *HSPA4L*, *ACSS2*, *C4BPA*, *C4BPA*, *AHSA1*, *PPP1CC*, *PPP1CA*, *ACSL3*, *MARCH2*, *BPIFC*, *VEGFB*, *SLAMF7*, *GPX2*	*DAB2*, *LOC100517400*, *CEBPB*, *STAT2*, *STAT1*, *STAT3*, *UBASH3B*, *LOC100518083*, *LOC100626135*, *TNS4*, *ACSL4*, *CEBPB*, *LITAF*, *GAB2*, *STAT5A*, *OASL*, *CREB3L2*, *TNFSF10*, *GPX8*, *LOC100623062*, *ACER2*, *MARCH3*, *ALOX12*, *LOC100156301*, *CEBPD*, *PLEKHG3*, *APOH*, *STAT2*, *FAP*
	ProxUt	*GAB1*, *HSPB1*, *HAGHL*, *HSPH1*, *LMX1A*, *LMX1B*, *CD1D*, *PTGS1*, *MARCH2*, *PLA2G4D*, *VEGFB*	*DAB2*, *STAT2*, *STAT1*, *MCF2*, *STAT3*, *UBASH3B*, *LOC100518083*, *ACSL4*, *LITAF*, *STAT5B*, *STAT5A*, *OASL*, *CREB3L2*, *CD38*, *TNFSF10*, *GPX8*, *LOC100623062*, *SELP*, *MARCH3*, *SLA-7*, *PLEKHG3*, *FAP*
	UTJ	*DUOX2*, *LOC100518524*, *VEGFB*, *GAB1*, *CD84*, *SLAMF6*, *AIF1*, *CD2*, *ACSL3*, *HVCN1*, *GIMAP8*	*CEBPB*, *CD209*, *PRKAB2*, *ACSBG1*, *TNS4*, *CEBPB*, *LITAF*, *STAT5A*, *CREB3L2*, *GPX8*, *ADGRB2*, *TRAF2*, *PLEKHG3*, *VEGF*, *FAM43A*, *FAP*
	Isth	*GAB1*, *SLAMF7*, *ACSS1*, *SIT1*, *MARCH2*, *HVCN1*, *WFIKKN2*, *SLA-6*	*SLC27A4*, *ACSBG1*, *LITAF*, *STAT5A*, *GPX8*
	Amp	*DUOX2*, *LOC100518524*, *VEGFB*, *LOC100517400*, *GAB1*, *HSPB1*, *STAT3*, *DPP4*, *LOC100626135*, *TNS4*, *SIT1*, *ADGRD2*, *ACER2*, *CD1E*, *DLL3*, *LOC100156301*, *PTGS1*, *CEBPD*, *CEBPE*, *GIMAP8*, *WFIKKN2*, *SLA-6*	*KIAA0319*, *LOC100518083*, *WEE1*, *HSPA4L*, *PGF*, *GPX8*, *AIFM3*, *ADGRB2*
	Inf	*LOC100517400*, *GAB1*, *SLAMF7*, *LY9*, *STAT3*, *CELSR1*, *LOC102161621*, *LOC100626135*, *TNS4*, *ACSS2*, *GAB2*, *TNFSF10*, *LOC100156301*, *CEBPD*, *VEGFA*, *WFIKKN2*, *LOC100518524*, *VEGFB*, *CD84*, *SLAMF6*	*MAPK8IP1*, *ACSBG1*, *HSPA4L*, *AHSA1*, *STAT5A*, *PGF*, *GPX8*, *AIFM3*, *ADGRB2*, *CSRP2*, *MARCH2*, *PRDX4*, *TRAF3*, *DLL4*
P1-AI	Cvx	*LOC100739854*, *GPX5*, *ACER2*, *GIMAP8*, *DAB1*	*HSPA4L*
	DistUt	*LMX1A*, *BPIFC*, *SLA-6*	*TNS4*, *CREB3L2*, *ALOX12*, *PTGS1*, *PLEKHG3*
	ProxUt	*DUOX2*, *SLAMF6*, *TNFSF11*, *LMO4*, *SLC11A1* , *SIT1*, *LOC100513601*, *CD1D*, *BPIFC*, *HVCN1*, *CD244*, *SLA-6*	*MCF2*, *UBASH3B*, *STAT5B*, *GPX8*
	UTJ	*LOC100518524*, *VEGFB*, *GAB1*, *GPX5*, *CRYAB*, *DPP4*, *ACSL1*, *CDIP1*, *MARCH2*, *WFIKKN2*	*CXCL16*, *CELSR1*, *LOC100518083*, *TNS4*, *ACSL4*, *HSPA4L*, *VAV2*, *CD38*, *ACER2*, *CSRP2*, *PLEKHG3*, *VEGFA*, *VAV1*
	Isth	*VEGFB*, *GAB1*, *LOC100626135*, *GAB2*, *LOC100156301*, *BPIFC*, *SLA-6*, *FAP*	*HSPH1*, *PPP1CA*
	Amp	*VEGFB*, *HEPACAM*, *LOC100513601*, *DLL3*, *BPIFC*, *ADGRB2*, *STAT1*	*HSPA4*, *LOC100518083*, *HSPH1*, *OASL*
	Inf	*VEGFB*, *LOC100517400*, *GAB1*, *LOC102161621*, *HEPACAM*, *ACSS2*, *C4BPA*, *GAB2*, *C4BPA*, *ACER2*, *LOC100513601*, *BPIFC*	*DUOX1*, *HSPA4*, *HSPH1*, *AHSA1*, *SLC11A1*, *OASL*, *PGF*, *GPX8*, *ADGRB2*, *CSRP2*, *CEBPE*, *LTA*
SP-Ejac	Cvx	*ACSS2*, *C4BPA*, *ACER2*, *DAB1*	*UBASH3B*, *CREM*, *ACSBG1*, *TNS4*, *MARCH3*, *APOH*
	DistUt	*HSPA4L*, *PPP1CC*, *PRDX4*, *BPIFC*, *DAB1*	*CEBPB*, *SLC27A4*, *GPX5*, *UBASH3B*, *TNS4*, *CEBPA*, *SIT1*, *STAT5A*, *MARCH3*, *LOC100156301*, *CEBPD*, *PLEKHG3*, *APOH*
	ProxUt	*PRKAB2*	*DUOX2*, *MARCH3*
	UTJ		*CEBPB*, *ACSBG1*, *TNS4*, *CEBPB*, *ADGRD2*, *TRAF2*
	Isth	*PCD1B*, *TNS4*, *PLA2G4D*	
	Amp	*UBASH3B*	
	Inf	*SELP*, *MZB1*	*GPX2*, *HAGHL*, *CEBPE*
SP-P1	Cvx	*ACER2*, *LOC100513601*	*GAB2 APOH*
	DistUt	*LOC102161621*, *HSPA4L*, *LMX1A*, *PPP1CC*, *BPIFC*	*CEBPB*, *LY9*, *SLC27A4*, *TNFSF14*, *LOC100626135*, *LTB*, *TNS4*, *CEBPB LMO2*, *LTB*, *SIT1*, *MARCH3*, *LOC100156301*, *PTGS1*, *CEBPD*, *PLEKHG3*
	ProxUt	*LOC100513601*, *CD1D*	*SELE*, *LMO2*, *SELE*, *GPX8*, *ADGRB2*, *MARCH3*
	UTJ	*TNFSF4*, *LOC100513601*, *BPIFC*, *GIMAP8*, *WFIKKN2*	*CEBPB*, *STAT3*, *TNS4*, *ADGRD2*, *TRAF2*, *CEBPD*, *CEBPE*
	Isth	*PCD1B*, *DPP4*, *DPP10*, *FAP*	*SLC27A4*, *MAPK8IP1*, *LOC100518083*, *PPP1CA*, *VAV1*
	Amp	*LMX1A*, *LOC100513601*, *APOH*, *BPIFC*	*STAT1*, *LOC100518083*, *OASL*
	Inf	*LOC102161621*, *SELP*, *LOC100513601*, *FAP*	*ACSBG1*, *STAT5A*, *OASL*, *ADGRB2*, *FAM43A*

**Table 2:** Immune-process genes (FDR/PCA, including Kyoto Encyclopedia of Genes and Genomes (KEGG) pathway/s) displaying changes in expression in specific segments (Cvx: cervix, DistUt: distal uterus, ProxUt: proximal uterus, UTJ: Utero-tubal junction, Isth: isthmus, Amp: ampulla or Inf: infundibulum) of the pig estrous internal genital tract, 24 h after mating or cervical deposition of the sperm-peak portion (P1-AI), of its SP (SP-P1) or SP from the whole ejaculate (SP-Ejac).

Gene	KEGG-Pathway	FDR *q* < 0.05	PCA *p* < 0.003	Up-Regulated (red) or Down-Regulated (green) per Tissue Segment after Treatment (mating, P1-AI, SP-Ejac, SP-P1)						
				Cvx	DistUt	ProxUt	UTJ	Isth	Amp	Inf
*AIFM3*	Endocrine resistance	YES	YES						**Mating** *	**Mating** *
*APOH*	Cholesterol metabolism	YES*	YES		**Mating** *				**SP-P1**	
*CD209*	C-type lectin receptor signaling pathway	-	YES	**Mating**						
*CD244*	Natural killer cell mediated cytotoxicity	-	YES			**P1-AI**				
*CREB3L2*	TNF, AMPK, cAMP, PI3K-Akt and cGMP-PKG signaling pathways	YES	-		**Mating** *	**Mating** *				
*CRYAB*	Protein processing in endoplasmic reticulum	-	YES			**P1-AI**	**P1-AI**			
*DPP4*	Protein digestion and absorption	-	YES					**SP-P1**		
*FAP*	JAK-STAT signaling pathway, apoptosis	YES*	YES		**Mating**	**Mating** *				
*GAB1*	Bacterial invasion of epithelial cells, Ras signaling pathway	YES*	YES					**P1-AI**		**Mating** *
*GAB2*	Fc epsilon RI and Fc gamma R-signaling pathways	-	YES					**P1-AI**		
*GPX2*	Arachidonic acid and Glutathione metabolism	YES	-		**Mating** *					
*GPX5*	Arachidonic acid and Glutathione metabolism	-	YES		**Mating**					
*GPX8*	Arachidonic acid and Glutathione metabolism	YES	YES		**Mating** *	**Mating** *	**Mating** *			**Mating**
*HSPA4L*	Protein processing in endoplasmic reticulum	YES*	YES			Mating */ SP-P1^♯^				
*LOC100156301*	---	YES	-							**Mating**
*LOC100518524*	---	-	YES				**P1-AI**			
*ADGRB2*	Cell surface receptor signaling and G-protein coupled receptor activity	YES*	YES						**Mating** *	Mating */ P1-AI
*LOC100739854*	---	-	YES	**P1-AI**						
*LOC102161621*	---	YES	YES							**Mating**
*OASL*	Human papillomavirus infection	-	YES			**Mating**				
*PGF*	MAPK, PI3K-Akt, Rap1 and Ras signaling pathways	YES	YES							**Mating** *
*PLEKHG3*	---	YES	-		**Mating** *					
*PRDX4*	---	YES	-							**Mating** *
*STAT5A*	JAK-STAT signaling pathway, Th1, Th2 and Th17cell differentiation	YES	-		**Mating** *			**Mating** *		
*TNFSF10*	Cytokine-cytokine receptor interaction, Apoptosis, FoxO signaling pathway, Natural killer cell mediated cytotoxicity	-	YES							**Mating/** **P1-AI**
*TNFSF11*	Cytokine-cytokine receptor interaction, NF-kappa B signaling pathway	-	YES				**Mating**			
*UBASH3B*	---	YES	YES	**Mating** *	**Mating** *	**Mating** *				
*VAV2*	Leukocyte transendothelial migration, B cell receptor, Chemokine, cAMP, Rap1, Fc epsilon RI signaling pathways, Fc gamma R-mediated phagocytosis, Natural killer cell mediated cytotoxicity, T cell receptor signaling pathway	-	YES				**P1-AI**			
*WFIKKN2*	Prolactin signalling pathway	-	YES				**P1-AI**			

* FDR values were only obtained in Mating, but not in the other treatments.

## Supplementary Materials

Supplementary materials can be found at http://www.mdpi.com/1422-0067/20/3/513/s1.
