# Supplementary material for: Expression of Immune Regulatory Genes in the Porcine Internal Genital Tract Is Differentially Triggered by Spermatozoa and Seminal Plasma
Source: Int J Mol Sci. 2019 Jan 25;20(3):513. doi: 10.3390/ijms20030513 (PMC6387272; doi:10.3390/ijms20030513)
Supplement: Supplementary file 1 [file ijms-20-00513-s001.pdf]

Supplementary Table S1. Principal Component analysis (PCA) of up- (positive logFC) or down (negative logFC)-regulated annotated genes involved in immune processes found in the different segments of the sow genital tract (cervix-to-infundibulum), after the various treatments (Mating, P1-AI, SP-Ejac and SP-P1) were compared to Control. Those genes with coloured p-corrected values showed significant differences (p<0.003), while the rest account for suggestive differences (p<0.01). Treatments= Mating: natural breeding P1-AI: inseminated with sperm from the sperm-rich fraction, SP-Ejac: cervical infusion with SP from the entire ejaculate, SP-P1: cervical infusion with SP from the sperm-peak fraction (P1). Tissue= CVx: Cervix, DistUt: distal uterus, ProxUt: proximal uterus, UTJ: utero-tubal junction, Isth: isthmus, Amp: Ampulla, Inf: Infundibulum. Red data: up-regulated; green data: down-regulated.

Treatment	Tissue	Gene	logFC	AveExpr	t	P.Value
Mating	Cvx	*CD209*	-0,3831354	2,365705	-4,1656	0,002660586
Mating	Cvx	*DAB2*	-0,1401652	2,973202	-3,278139	0,010114616
Mating	Cvx	*DUOX2*	-0,2642187	2,324291	-3,571891	0,006427954
Mating	Cvx	*FAP*	-0,2027853	3,001545	-4,072504	0,003045379
Mating	Cvx	*GPX8;_LOC100515061*	-0,1888078	2,744798	-3,273983	0,010180413
Mating	Cvx	*LOC100518083*	-0,245974	3,015298	-3,312363	0,00958935
Mating	Cvx	*SLC11A1*	-0,1409802	2,207932	-3,592492	0,006229243
Mating	Cvx	*STAT5A*	-0,1449493	2,578175	-3,456831	0,007667583
Mating	Cvx	*UBASH3B*	-0,2054032	2,81404	-4,502521	0,001648993
Mating	DistUt	*APOH*	-0,20930347	1,286938	-5,581595	0,000392705
Mating	DistUt	*APOH*	-0,1214636	1,330858	-3,30582	0,009429449
Mating	DistUt	*CREB3L2*	-0,14086663	3,009257	-3,899417	0,003899374
Mating	DistUt	*FAP*	-0,23411857	2,944698	-3,386266	0,008503777
Mating	DistUt	*GPX2*	0,28190419	1,975354	3,27588	0,010099047
Mating	DistUt	*GPX8;_LOC100515061*	-0,28131254	2,62834	-5,190362	0,00064591
Mating	DistUt	*HSPA4L*	0,16621015	2,801872	3,43727	0,007858027
Mating	DistUt	*LOC100156301*	-0,09913426	2,722507	-3,286464	0,009933348
Mating	DistUt	*LOC100517400*	-0,16907913	2,481763	-3,28913	0,009892063
Mating	DistUt	*OASL*	-0,34972435	2,419629	-4,267703	0,002277945
Mating	DistUt	*PLEKHG3*	-0,16408576	2,811708	-3,662087	0,005568915
Mating	DistUt	*STAT5A*	-0,26596412	2,548826	-3,552752	0,006578951
Mating	DistUt	*UBASH3B*	-0,22205163	2,751042	-4,693803	0,001253342
Mating	ProxUt	*CREB3L2*	-0,12782755	2,968809	-3,444172	7,67E-03
Mating	ProxUt	*FAP*	-0,26864584	2,98053	-4,134875	2,71E-03
Mating	ProxUt	*GPX8;_LOC100515061*	-0,40934656	2,639462	-6,890757	8,24E-05
Mating	ProxUt	*OASL*	-0,34443406	2,413484	-3,731733	4,93E-03
Mating	ProxUt	*SELP*	-0,21818817	2,426041	-3,333421	9,12E-03
Mating	ProxUt	*UBASH3B*	-0,27005612	2,723995	-3,694248	5,22E-03
Mating	UTJ	*AIF1*	0,17730355	2,488033	3,365879	0,007906626
Mating	UTJ	*CD209*	-0,22434791	2,628941	-3,796674	0,003975752
Mating	UTJ	*GIMAP8*	0,18090861	2,109859	3,249428	0,009554398
Mating	UTJ	*GPX8;_LOC100515061*	-0,16388094	2,97626	-4,211192	0,002097597
Mating	UTJ	*LOC100739244;_ADGRB2*	-0,12304311	2,324522	-3,654288	0,004978013
Mating	UTJ	*TNFSF11*	-0,18056245	1,632456	-5,302144	0,000437865
Mating	UTJ	*VEGFA*	-0,32893766	2,247762	-3,707532	0,004575286
Mating	Isth	*STAT5A*	-0,14992077	2,594985	-3,473329	0,007423557
Mating	Amp	*AIFM3*	-0,16856304	2,596608	-4,247979	0,002289509
Mating	Amp	*DLL3*	0,12918834	2,057887	3,675742	0,005359699
Mating	Amp	*LOC100739244;_ADGRB2*	-0,25614354	2,361015	-5,529389	0,000404227
Mating	Inf	*AIFM3*	-0,13691738	2,602532	-3,961051	0,004588554
Mating	Inf	*CD84*	0,27373616	1,789405	3,455943	0,009276025
Mating	Inf	*GAB1*	0,16722325	2,786663	3,747751	0,006149324
Mating	Inf	*GPX8;_LOC100515061*	-0,15657203	2,737895	-3,987331	0,00442803
Mating	Inf	*LOC100156301*	0,11001431	2,522069	3,639518	0,007152208
Mating	Inf	*LOC100739244;_ADGRB2*	-0,37349369	2,285485	-5,487399	0,000685958
Mating	Inf	*LOC102161621*	0,49766726	2,463593	4,714296	0,001722029
Mating	Inf	*PGF*	-0,25509557	2,414271	-4,369276	0,002669717
Mating	Inf	*PRDX4*	-0,16340768	2,981712	-3,662006	0,006930232
Mating	Inf	*TNFSF10*	0,32104087	2,637521	6,350584	0,00026955

P1-AI	Cvx	*CD244*	0,1339507	2,200656	3,316436	0,009629736
P1-AI	Cvx	*LOC100739854*	0,2701084	1,054534	4,19967	0,002576031
P1-AI	DistUt	*ALOX12*	-0,14491567	2,122224	-3,74985	0,004909484
P1-AI	DistUt	*PTGS1*	-0,12667916	2,078113	-3,249659	0,010574195
P1-AI	ProxUt	*BPIFC*	0,21253228	2,0579092	3,4263442	0,008631481
P1-AI	ProxUt	*CD1D*	0,10275171	2,3995802	3,4177114	0,00874404
P1-AI	ProxUt	*CD244*	0,15751764	2,0697973	4,5076516	0,00184687
P1-AI	ProxUt	*CRYAB*	-0,09649741	2,3364335	-4,1629732	0,002964783
P1-AI	ProxUt	*SLC11A1*	0,16217971	2,2340857	3,4376229	0,008486722
P1-AI	UTJ	*ACER2*	-0,20810282	2,760579	-3,5584059	0,006618556
P1-AI	UTJ	*CRYAB*	0,21201316	2,89385	4,5666735	0,001529297
P1-AI	UTJ	*LOC100518524*	0,23849731	2,615886	4,1798891	0,002637253
P1-AI	UTJ	*MARCH2*	0,17518838	2,740342	3,4531192	0,007774419
P1-AI	UTJ	*TNS4*	-0,19591435	2,544682	-3,9662076	0,003596891
P1-AI	UTJ	*VAV2*	-0,19046859	2,674336	-5,2867148	0,000587649
P1-AI	UTJ	*VEGFA*	-0,23021724	2,313567	-3,3997981	0,008439043
P1-AI	UTJ	*WFIKKN2*	0,18796128	2,089891	4,2477526	0,002393007
P1-AI	Isth	*GAB1*	0,10182262	2,860111	4,194552	0,002763872
P1-AI	Isth	*GAB2*	0,11102011	2,842033	5,039555	0,000888295
P1-AI	Isth	*HSPH1*	-0,0752612	3,221459	-3,458073	0,008084167
P1-AI	Isth	*PTGS1*	0,09795044	2,235353	3,608712	0,006451778
P1-AI	Isth	*SLAMF7*	0,14620364	1,917992	3,655108	0,006022382
P1-AI	Amp	*LOC100517400*	0,11259252	2,4262135	3,8551976	0,003935117
P1-AI	Amp	*OASL*	-0,27541553	2,5446392	-3,5936457	0,005883188
P1-AI	Inf	*BPIFC*	0,22011925	2,4406853	3,6471968	0,005197168
P1-AI	Inf	*C4BPA*	0,21677276	2,6930867	3,2378599	0,009979867
P1-AI	Inf	*CEBPE*	-0,15925149	1,8130225	-3,3381088	0,008491913
P1-AI	Inf	*DUOX1*	-0,16103504	1,963051	-3,4105091	0,007562081
P1-AI	Inf	*GAB1*	0,12751932	2,7787554	3,5313412	0,006239353
P1-AI	Inf	*HEPACAM*	0,12731739	2,6865922	3,9050401	0,003481192
P1-AI	Inf	*LOC100739244;_ADGRB2*	-0,32672787	2,2821899	-4,6633065	0,001129436
P1-AI	Inf	*LOC102161621*	0,36790213	2,4342577	3,833849	0,003885146
P1-AI	Inf	*TNFSF10*	0,20407199	2,6019676	5,0812475	0,000629175
SP-Ejac	Cvx	*DAB1*	0,2139356	1,9146745	3,633497	0,005726152
SP-Ejac	DistUt	*GPX5*	-0,51037	0,461988	-4,712722	0,001169578
SP-Ejac	Inf	*CEBPE*	-0,13524	1,8250282	-3,577137	0,006168081
SP-Ejac	Inf	*LOC100739854*	-0,1519294	0,6510671	-3,470345	0,007278687
SP-P1	DistUt	*HSPA4L*	0,19229477	2,8149143	5,5848024	0,000429791
SP-P1	UTJ	*TNFSF4*	0,1703252	2,045535	4,119587	0,003312638
SP-P1	UTJ	*WFIKKN2*	0,1345113	2,066983	3,575686	0,007178162
SP-P1	Isth	*DPP4*	0,26734983	2,2993539	6,0092792	0,000263365
SP-P1	Amp	*APOH*	0,22284445	1,4694809	4,73485	0,001194489
SP-P1	Inf	*CEBPD*	-0,09605281	2,63641	-3,505493	0,00683823
SP-P1	Inf	*SELP*	0,11713319	2,4503334	3,297035	0,009484176

Gene: Full gene identification; logFC: log Fold Change of the treatment relative with control; AveExpr: average expression of the probe sets; t: is the ratio of the estimated value of a parameter from its hypothesized value to its standard error and P-Value: p-value from the t-test.

Supplementary Table S2. Number of genes belonging to different pathways of immune function processes (GO:0002376) up- (U) or down (D)-regulated found in the different segments of the sow genital tract (cervix-to-infundibulum), after the various treatments (Mating, P1-AI, SP-Ejac and SP-P1) were compared to Control.

Pathway	Cvx								DistUt								ProxUt								UTJ								Isth								Amp								Inf							
	Mating		P1-AI		SP-Ejac		SP-P1		Mating		P1-AI		SP-Ejac		SP-P1		Mating		P1-AI		SP-Ejac		SP-P1		Mating		P1-AI		SP-Ejac		SP-P1		Mating		P1-AI		SP-Ejac		SP-P1		Mating		P1-AI		SP-Ejac		SP-P1		Mating		P1-AI		SP-Ejac		SP-P1	
	U	D	U	D	U	D	U	D	U	D	U	D	U	D	U	D	U	D	U	D	U	D	U	D	U	D	U	D	U	D	U	D	U	D	U	D	U	D	U	D	U	D	U	D	U	D	U	D	U	D	U	D	U	D	U	D
Acetate utilization (P02722)					1				1																								1																1		1					
Apoptosis signaling pathway (P00006)						1				1						2		1								1				1	1	1																	1			1				
CCKR signaling map (P06959)						1				1							1	2										1				1									2								1							
Enkephalin release (P05913)						1																																																		
Heterotrimeric G-protein signaling pathway-Gi alpha and Gs alpha mediated pathway (P00026)						1				1		1						1								1					1																									
Transcription regulation by bZIP transcription factor (P00055)						1				1		1						1								1																														
EGF receptor signaling pathway (P00018)	1	8						1	1	10				3			1	10		1					1	3	1				3	1	1	3	2						2		3	3				3	3	3	2					3
PDGF signaling pathway (P00047)	1	8						1	1	10				3			1	10		1					1	3	1	2			3	1	1	3	2					1	2		3	3				3	3	3	2					3
Angiogenesis (P00005)		4								4							2	4								1	1	1				1					1				3		4	3				3	2	1						
Inflammation mediated by chemokine and cytokine signaling pathway (P00031)		3								5		2				1	1	4										1				1								1	2		3	3				3	1			1				
Interferon-gamma signaling pathway (P00035)		3								3								3																									3	3				3	1							
Interleukin signaling pathway (P00036)		8								9				3				10		1						3					3	1		3							1		3	3				3	1	3						3
JAK/STAT signaling pathway (P00038)		8								9				3				10		1						3					3	1		3							1		3	3				3		3						3
Notch signaling pathway (P00045)		1																																							1		1							1						
Oxidative stress response (P00046)		3								3								3																									3	3				3								
Ras pathway (P04393)		3								4								4														1									1		3	3				3	1							
p53 pathway feedback loops 2 (P04398)		3								3								3																								1	3	3				3								
Gonadotropin-releasing hormone receptor pathway (P06664)										1								1			1					1					1	1									1								1							
VEGF signaling pathway (P00056)																	1									1	1	1													1								1							
p38 MAPK pathway (P05918)																	1																								1															
p53 pathway (P00059)																								1		2				1	2	1											1							1		1				1
Dopamine receptor mediated signaling pathway (P05912)									2				1		1																					1				1																
Nicotine pharmacodynamics pathway (P06587)									2				1		1																					1				1																
B cell activation (P00010)										1								1										2												1																
T-cell activation (P00053)										1								1										2												1																
Wnt signaling pathway (P00057)										1								1																																						
p53 pathway by glucose deprivation (P04397)																					1					1					1																									
UP-REGULATED (%)	2 (3.7)		0 (0)		1 (16.7)		0 (0)		7 (9.3)		0 (0)		2 (14.3)		2 (40)		8 (10.3)		0 (0)		2 (100)		0 (0)		2 (8.7)		4 (28.6)		0 (0)		18 (62.1)		3 (20)		4 (66.7)		1 (100)		0 (0)		18 (94.7)		33 (52.4)		0 (0)		0 (0)		17 (53.1)		5 (62.5)		0 (0)		0 (0)	
DOWN-REGULATED (%)	52 (96.3)		0 (0)		5 (83.3)		2 (100)		68 (90.7)		4 (100)		12 (85.7)		3 (60)		70 (89.7)		4 (100)		0 (0)		1 (100)		21 (91.3)		10 (71.4)		2 (100)		11 (37.9)		12 (80)		2 (33.3)		0 (0)		6 (100)		1 (5.3)		30 (47.6)		0 (0)		30 (100)		15 (46.9)		3 (37.5)		0 (0)		13 (100)	
